# Causes of Intermittent and Remittent Fever

**Published:** 1866-06

**Authors:** 


### CAUSES OF INTERMITTENT AND REMITTENT FEVER.

Prof. J. H. Salisbury communicates to the *American Journal of Medical Sciences* an elaborate article, giving an account of numerous observations and investigations regarding the origin and cause of intermittent fever. Dr. Salisbury found on microscopic examination of the salivary secretion and expectoration of those laboring under intermittent fever, and who resided upon ague levels, and were exposed to the evening, night, and morning exhalations and vapors arising from stagnant pools, swamps, and humid low grounds, that there occurred in these secretions a great variety of zoosporoid cells, animalcular bodies, diatoms, dismidiæ, algoid cells and filaments, and fungoid spores. Constantly and uniformly found in all cases, and usually in great abundance, were minute oblong cells, either single or aggregated, consisting of a distinct nucleus, surrounded by a smooth cell-wall, with a highly clear, apparently empty space between the outside cell-wall and the nucleus. They were fungoid, but cells of an algoid type, resembling strongly those of the *palmellæ*. In persons residing *above* the summit plane of ague, the bodies were invariably absent.

By a series of carefully conducted experiments and observations, the following facts were ascertained:—

1. That cryptogamic spores, and other minute bodies, are mainly elevated above the surface during the night. That they rise and are suspended in the cold damp exhalations from the soil, after the sun has set, and fall again to the earth soon after it rises.

2. That in the latitude of Ohio, these bodies seldom rise above from thirty-five to sixty feet above the low levels. In the northern and central portions of the State they rise from thirty-five to forty-five feet, while in the southern, from forty to sixty feet.

3. That at Nashville and Memphis they rise from sixty to one hundred feet and more above the surface.

4. That above the summit plane of the cool night exhalations, these bodies do not rise, and intermittents do not extend.

5. That the day air of malarial districts is quite free from these palmelloid spores, and from causes that produce intermittents.

*Palmellæ* belong to the lowest known vegetable organisms. The several forms of this type which are constantly attendant on intermittent malarial districts, have received the generic name *gemiasma* (earth miasma), of which Dr. Salisbury enumerates six species.

In another series of extended observations, the local effects produced in the mouth and air-passages by inhaling these cells, are minutely described. They cause a dry, feverish, constructed feeling in the mouth, fauces, and throat, increasing until the fauces become parched and feverish, normal mucous discharges become checked, and the feeling soon extends to the bronchial and pulmonary surfaces, which also become dry, feverish, and constricted, with a heavy congested sensation and dull pain. These peculiar symptoms generally last several hours after leaving the bog.

The author has made experiments relative to the production of intermittent fever in localities entirely free from malarial influence, by carrying boxes filled with surface earth from a malarious drying prairie bog, covered with the *palmellæ*, to these localities, and exposing persons to their emanations. Attacks of intermittent were the result.

The investigations of Dr. Salisbury must be considered highly important, as they establish positively the *fons et origo* of malarious fever.

Dr. E. Holdon, of Newark, N.J., late of the U.S.N., communicates a paper to the same journal, entitled, "An Inquiry into the Causes of certain Diseases on Ships of War," in which he expresses his opinion that fever of an intermittant type is produced by the growth of mould on board of ship, under the action of hydro-sulphuric acid of the bilge.—*Med. and Surg. Reporter.*

